# *BARCRAWL *and *BARTAB*: software tools for the design and implementation of barcoded primers for highly multiplexed DNA sequencing

**DOI:** 10.1186/1471-2105-10-362

**Published:** 2009-10-29

**Authors:** Daniel N Frank

**Affiliations:** 1 Department of Molecular, Cellular and Developmental Biology, University of Colorado, Boulder, CO, 80309, USA; 2 Mucosal and Vaccine Research Program, Colorado, USA

## Abstract

**Background:**

Advances in automated DNA sequencing technology have greatly increased the scale of genomic and metagenomic studies. An increasingly popular means of increasing project throughput is by multiplexing samples during the sequencing phase. This can be achieved by covalently linking short, unique "barcode" DNA segments to genomic DNA samples, for instance through incorporation of barcode sequences in PCR primers. Although several strategies have been described to insure that barcode sequences are unique and robust to sequencing errors, these have not been integrated into the overall primer design process, thus potentially introducing bias into PCR amplification and/or sequencing steps.

**Results:**

*Barcrawl *is a software program that facilitates the design of barcoded primers, for multiplexed high-throughput sequencing. The program *bartab *can be used to deconvolute DNA sequence datasets produced by the use of multiple barcoded primers. This paper describes the functions implemented by *barcrawl *and *bartab *and presents a proof-of-concept case study of both programs in which barcoded rRNA primers were designed and validated by high-throughput sequencing.

**Conclusion:**

*Barcrawl *and *bartab *can benefit researchers who are engaged in metagenomic projects that employ multiplexed specimen processing. The source code is released under the GNU general public license and can be accessed at .

## Background

Recent developments in culture-independent microbiology now permit "metagenomic" analyses of complex microbial communities in which mixtures of genes or genomes are sequenced and analyzed in parallel [1,2]. Concomitant advances in automated DNA sequencing technology have greatly increased the scale of genomic and metagenomic studies [3]. In many instances, project throughput can be accelerated by parallelization of sample processing, sequencing, and analysis steps. High-throughput sequencing platforms now have the capacity to analyze multiple specimens in a single run. Such multiplexing can be accomplished either through physical separation of samples on the sequencing instrument (e.g. splitting samples across 16 cells on the 454 Life Sciences GS-FLX system [4]) or by tagging genomic DNAs with unique, sample-specific sequences that serve as molecular barcodes [5,6].

Two strategies are in general use for barcoding samples in preparation for high-throughput sequencing. In one approach, barcoded, double-stranded adapters are ligated to target DNA through the activity of DNA ligase; both shotgun genomic libraries and PCR amplicons can be tagged in this manner [7]. The second strategy, the topic of this paper, incorporates barcodes into oligonucleotides that are used to prime DNA synthesis, for instance, through polymerase chain reaction (PCR; [5,6]). Typically, gene-specific oligonucleotides are used to amplify and tag a particular gene, for instance the ribosomal RNA gene (rDNA).

Because DNA ligation is relatively sequence-independent, the design of barcoded adapters using the ligated adapter strategy is straightforward. In contrast, proper design of barcoded primers requires careful consideration of how the inclusion of barcode sequences (and any other required sequences e.g., the "A" and "B" sequences required by the 454 GS-FLX platform) may impact the specificity, sensitivity, and overall efficiency of primer-dependent steps of the workflow. These design issues have motivated the creation of a computer program, *barcrawl*, for automated design of barcoded primer pairs. *Barcrawl *constructs a set of barcodes, each separated in sequence space from all other barcodes by a minimum number of base substitutions (default value of 3 events). Barcodes are evaluated within the context of other sequences contained in the forward and reverse PCR primers in order to cull potentially problematic sequences (e.g., due to inhibitory homopolymers, potential hairpins or heteroduplex formation between primers). Finally, *barcrawl *sorts the set of barcodes by the number of 454 GS-FLX nucleotide flows required to pyrosequence each barcode sequence. Use of more efficiently sequenced barcodes (i.e., those with minimal flow patterns) should help maximize read lengths within the template regions of amplicons. To facilitate manipulation of barcoded sequence data sets following multiplexed sequencing, we present the program *bartab*, which is a general purpose tool for polishing and annotating sequence data sets. Source code for both *barcrawl *and *bartab *is freely available for use under the GNU General Public License at . Pre-compiled, universal binaries for the Macintosh platform are provided in the Mac OS-X GUI package XplorSeq [8].

## Implementation

*Barcrawl *and *bartab *are command-line executables written in C++ and available as source code for compilation on multiple platforms. The software was developed on a Macintosh OS-X (10.5.5) system, using Xcode and gcc version 4.0.1 (Apple Inc. build 5484) and has been tested on Macintosh OS-X (10.4.x and 10.5.x) and multiple Linux platforms (e.g. Fedora, Ubuntu).

## Results and discussion

The following sections outline the algorithms used by *barcrawl *to design primers and *bartab *to post-process multiplexed DNA data sets. These are followed by an experimental test-case that outlines the design and validation of a set of 96 barcoded primers for broad-range PCR amplification of bacterial large subunit ribosomal RNA genes (LSU rDNA).

### Barcrawl Algorithm and Operation

**Figure 1:**
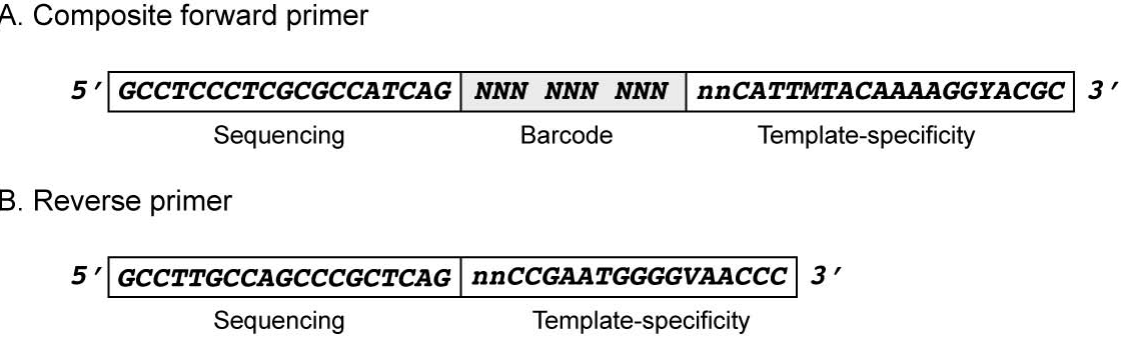
**Barcoded primer structures**. Design of barcoded oligonucleotides. "Barcode" indicates the location of sequences unique to each PCR primer. "Template-specificity" indicates sequences required for PCR amplification. In this example, template-specificity regions are broadly specific for bacterial large-subunit rRNA genes (A: LSU559R, B: LSU130F) and are joined to the rest of the primer through a two-nucleotide, randomized linker. Sequences labeled "Sequencing" refer to the primer A and B sequences required by the GS-FLX instrument. Although this example is based on specifications required for operation of the 454 LifeSciences Inc. GS-FLX system, the use of *barcrawl *can be abstracted to other platforms capable of sequencing PCR amplicons. The "sequencing" segments may not be required by other platforms and are not required for *barcrawl *to function.

In overview, *barcrawl *creates a list of all possible DNA sequences of a specified length and then progressively culls sequences that may interfere with primary PCR amplification and/or sequencing steps (i.e. homopolymers, hairpins, and/or heteroduplex formation). The primary structures of forward and reverse PCR primers used to barcode amplicons are schematized in Fig. 1. Regions labeled "Sequencing" in Fig. 1 are not required for *barcrawl *function, but are included in this example because they encode sequences necessary to perform direct pyrosequencing of amplicons on the 454 GS-FLX. At a minimum, the barcode must lie upstream of the specificity region of the oligonucleotide and in a position that can be sequenced by a particular platform. Well-designed barcode sequences must be robust to both mutation during amplification and errors in base-calling during sequencing. To this end, *barcrawl *performs an exhaustive pairwise comparison of all potential barcodes and discards those that differ in sequence compared to other barcodes by less than a user-specified cutoff value. The default value of 3 base differences insures that multiple substitution events or base-calling errors must occur for one barcode to be mis-classified as another.

**Figure 2:**
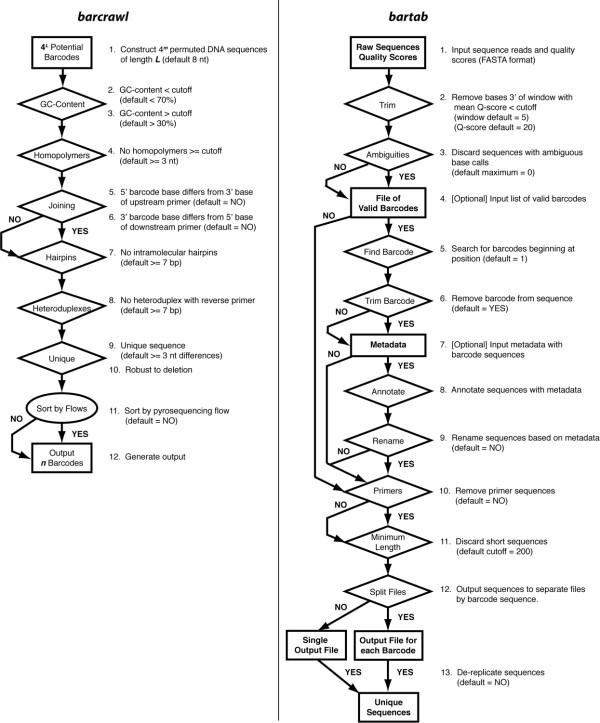
***Barcrawl *and *bartab *algorithms**. Rectangles specify input and output. Diamonds designate filters applied to data. Barcodes (*barcrawl*) or sequences (*bartab*) that do not follow the outlined rules are discarded. Arrows diverging from the central flow chart represent analytic steps that the user can choose to skip.

*Barcrawl *is invoked on the command line by typing *barcrawl [options]*. The default implementation requires no input from the user, although several options can be set on the command line (Table 1). Specific primer sequences to which the barcodes will be linked (either 5' or 3' to the barcode) along with a reverse primer sequence can be designated on the command line. The algorithm used by *barcrawl *to create and evaluate potential barcode sequences in the context of other primer sequences is as follows (Fig. 2):

**Table 1:** Summary of *barcrawl *command-line options.

**Option**	**Default**	**Min^1^**	**Max^2^**	**Effect**
-l <int>	= 8	1	20	Set length of barcodes

-m <int>	>= 3	1	barcode length	Minimum base substitutions between barcode pairs

-p <int>	>= 3	2	N	Exclude homopolymers of specified length or greater

-a <int>	>= 5	2	N	Exclude hairpins of specified length or greater

-b <int>	>= 5	2	N	Exclude heteroduplexes of specified length or greater

-f5 <string>	-	-	-	Specify 5' addition to barcode sequence

-f3 <string>	-	-	-	Specify 3' addition to barcode sequence

-j5	off	-	-	Exclude barcodes with 5' base same as 3' end of upstream primer

-j3	off	-	-	Exclude barcodes with 3' base same as 5' end of downstream primer

-r <string>	-	-	-	Specify reverse primer sequence

-g <int>	> 70	50	100	Exclude barcodes with % GC content greater than value

-c <int>	< 30	0	50	Exclude barcodes with % GC content less than value

-d	on	-	-	Exclude barcodes that are converted to other barcodes by deletion

-w	off	-	-	Order output by number of 454 GS-FLX nucleotide flows

-o <string>	out.txt	-	-	Specify output file

-v	off	-	-	Set verbose output to terminal

-h	off	-	-	Display help

^1^Minimum value for numerical options.

^2^Maximum value for numerical options. N = any non-negative integer.

1. For barcodes of length ***l ***(default = 8), generate 4∧f *l *strings encoding all permutations of the four standard DNA nucleobases (G, A, T, and C). Store strings in a list.

2. Remove from list all barcodes with G+C content > cutoff value (default > 70%).

3. Remove from list all barcodes with G+C content < cutoff value (default < 30%).

4. Remove from list all barcodes that contain homopolymers of length > = cutoff value (default = 3).

5. Concatenate potential barcode(s) to 5' and 3' sequences (if specified) to form composite forward primer. Remove from list all barcodes in which 5' base of barcode is identical to 3' base of upstream primer ("Sequencing" region in Fig. 1a).

6. Remove from list all barcodes in which 3' base of barcode is identical to 5' base of downstream primer ("Template-specificity" region in Fig. 1a).

7. Remove from list all barcodes for which composite forward primers potentially form intramolecular hairpins of length >= cutoff value (default = 7 basepairs).

8. Remove from list all barcodes for which composite forward primers potentially form heteroduplexes with reverse primer of length >= cutoff value (default = 7 basepairs).

9. For all pairwise combinations of barcodes, ***i ***and ***j***, remaining in list (i.e., those retained after steps 2-8), compute ***d***, the number of base differences between barcodes ***i ***and ***j***. Retain barcodes ***i ***and ***j ***if ***d ***>= cutoff value (default = 3 base differences), otherwise remove barcode ***j ***from list.

10. For all pairwise combinations of barcodes, ***i ***and ***j***, remaining in list (i.e., those retained after steps 2-9), determine whether any deletion within barcode ***j ***creates a string that is an exact match to a substring of ***i***. Determine whether any deletion within barcode ***i ***creates a string that is an exact match to a substring of ***j***. If an exact match is found in either search, then remove barcode ***j ***from list.

11. Sort list of remaining barcodes by number of nucleotide flows (*sensu *454 GS-FLX system) required to sequence through a primer.

12. Output list of barcodes, corresponding composite forward primers, and required pyrosequencing flows to tab-delimited file.

*Barcrawl *outputs the results of a search in two files: 1) A tab-delimited list of barcoded forward primers, the corresponding barcode sequences by themselves, and the number of pyrosequencing nucleotide flows required to sequence each barcode using the 454 GS-FLX system and 2) A logfile that summarizes the command options for a *barcrawl *run, statistics for number of barcodes output, distribution of flows for the set of barcodes, and elapsed execution time.

### Bartab Algorithm and Operation

The process of multiplexed high-throughput sequencing typically requires physical mixing of barcoded PCR libraries. Consequently, sequence reads generated with different barcoded primers must be identified and sorted into barcode-specific groups in order to associate sequences with the appropriate libraries. The command line software *bartab *provides a set of post-processing tools for checking sequence quality, identifying barcodes, annotating sequence reads, parsing sequences into barcode-specific groups, and de-replicating sequence sets (Fig. 2). While designed to complement the function of *barcrawl*, the two software programs function independently of one another.

**Figure 3:**
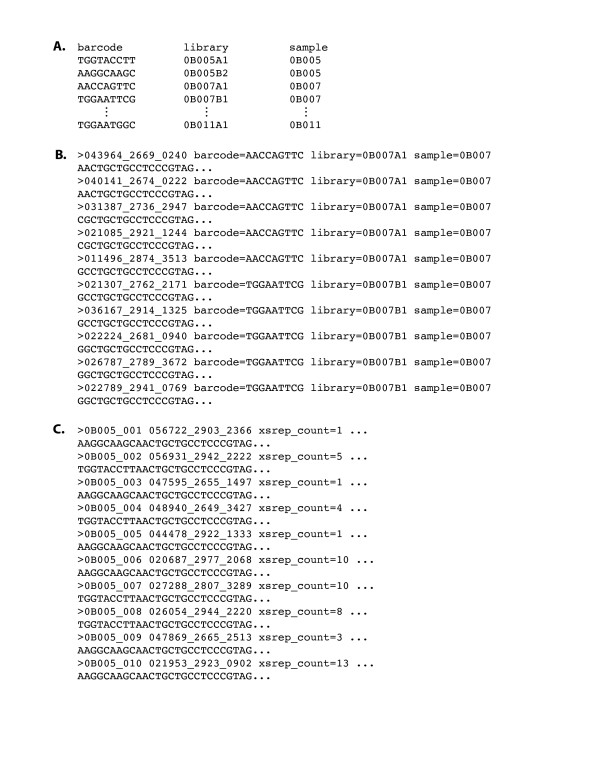
**Addition of Metadata through Barcode File**. A. Format of barcode file. Column headings act as keys for values in column cells. Strings specifying keys and values must consist of ASCII characters without whitespace. Values in a row are separated by tabs. B. Output of sequences annotated through metadata associated with barcodes. C. Output following sequence renaming and dereplication on the basis of metadata (in this case, by sample name). The "xsrep_count" metadata indicates the number of identical sequences recovered from a sample.

*Bartab *is invoked on the command line by typing *bartab [options]*. Command line options are summarized in Table 2. As input, *bartab *requires a FASTA formatted DNA sequence file and a corresponding file of quality scores in FASTA format. Optionally, a tab-delimited file that lists valid barcode sequences (one barcode per line) can be provided as input (example shown in Fig. 3a); sequences that do not encode a valid barcode are discarded if this file is present. The decision to discard sequences with invalid barcodes, rather than to infer the most likely barcode for a sequence, is based on the premise that sequences with mutated barcodes may encode other, non-correctable mutations in coding regions. This conservative approach does not unduly affect the total number of reads obtained from a sequencer run; we typically discard *ca*. 2% of sequences on the basis of invalid barcodes.

**Table 2:** Summary of *bartab *command-line options.

**Option**	**Default**	**Min**	**Max**	**Effect**
-in <string>	-	-	-	Fasta sequence file to process

-qin <string>	<fasta_file_name.qual>	-	-	Quality scores associated with sequences

-map <string>	<fasta_file_name.bar>	-	-	Tab delimited file listing barcodes and associated metadata

-out <string>	<fasta_file_name>	-	-	Base name for output files

-for <string>	-	-	-	Forward primer sequence

-rev <string>	-	-	-	Reverse primer sequence

-rnm <string>	off	-	-	Toggle on renaming of sequences based on column of barcode file named by specified string

-spl <string>	off	-	-	Toggle on splitting sequences into individual files based on column of barcode file named by specified string

-rep	off	-	-	Toggle on dereplication of output sequence file(s)

-st <int>	1	1	N	Position of barcode in sequence

-qu <int>	20	0	N	Minimum acceptable quality score, averaged over window

-win <int>	5	1	N	Window for calculation of mean quality score

-min <int>	200	1	N	Minimum acceptable sequence length

-amb <int>	0	0	N	Maximum acceptable number of ambiguous bases

-xbar	-	-	-	Toggle off removal of barcodes from sequences

-v	off	-	-	Verbose output to stdout

-dry	off	-	-	Dry run - report sequence statistics then quit

-h	off	-	-	Display help

^1^Minimum value for numerical options. N = any non-negative integer.

^2^Maximum value for numerical options.

Fig. 2 presents a flow chart of the main *bartab *functions. In overview, *bartab *applies a user-configurable series of filters to check the quality of each sequence read. These include filters based on minimum quality scores (default is an average Q-score of 20 across a window of 5 nts), presence of ambiguous base calls (default is to reject sequences with any ambiguities), removal of primer and barcode sequences, and minimum polished length above a threshold (default = 200 nt.).

In addition to validating barcodes, inclusion of a barcode file provides a ready means for both sorting sequences and annotating them with metadata that is keyed by barcode sequences. For instance, if unique barcodes are assigned to individual PCR libraries (e.g. from individual samples), then metadata specific to a library can be assigned to all of its constituent sequences. For this purpose, additional tab-delimited columns of data are included in the barcode file (Fig. 3a) in order to associate barcodes with metadata. Column headers specify keys (e.g., "barcode", "library", and "sample" in Fig. 3a), and data elements falling under a given column specify particular values to be associated with the barcode listed on the same row. A sequence can then be annotated with all key-value pairs associated with its barcode sequence. For ease of subsequent parsing, metadata are listed in FASTA definition lines using the format "key:value" (Fig. 3b). Such annotation is not mandatory. If a barcode file is not specified on the command line, *bartab *simply skips the barcode validation and sequence annotation steps (Fig. 2).

Polished sequences are output in FASTA format, as are corresponding quality scores. In addition, sequences (and quality scores) that are rejected based on their failure to satisfy the conditions of the filter steps, are discarded into a separate file (designated "<filename>.rej.fa" and "<filename>.rej.qual"). The default behaviour is to aggregate all acceptable, polished sequences into a single file. Alternatively, sequences assigned to different groups on the basis of barcode metadata can be split into multiple files by setting the "-spl <string>" option on the command line, where "<string>" denotes the name of a column in the barcode file. For instance, "-spl barcode" creates a separate output file for each unique barcode and then directs the output of polished sequences to the appropriate files based on their barcodes. Other annotation keys in the barcode file could also be selected as the basis of sorting. Analogously, selection of the rename option "-rnm <string>" replaces sequence names with a base name determined by the barcode file and a unique, sequential integer value for each sequence in the group (Fig. 3c).

Because high-throughput sequencing libraries may include multiple identical sequences, identifying, denoting, and removing such replicate sequences, can bring significant performance improvements to downstream applications. Toggling on the sort option ("-rep") causes *bartab *to de-replicate groups of like sequences. For each primary FASTA file that is output, *Bartab *uses a hash function to identify unique sequences (termed "sequence tags") and subsequently output the representative sequences to a FASTA file with the suffix "<filename>.rep.fa", for representative sequences. In order to preserve information about the relative abundances of sequences, *bartab *adds metadata to each representative sequence that specifies the number of sequences in the group (e.g. a barcoded library), designated by the key "xsrep_count" (Fig. 3c).

Lastly, *bartab *compiles detailed summary statistics, written either to a log file or the terminal, that include histograms and cumulative distributions of sequence length before and after trimming, as well as polished sequence counts for each barcode. *Bartab *can be executed in a dry run mode ("-dry") to tabulate these statistics without actually modifying sequence data. This feature allows the user to adjust input parameters if desired.

### Case Study: Application of Software to rRNA Metagenomics

In order to validate, refine, and demonstrate the use of *barcrawl *and *bartab*, a set of 96 barcoded oligonucleotides were designed and analyzed in a real world application. Barcoded-forward and reverse primers were constructed for broad-range PCR amplification of the bacterial LSU rDNA, using the highly-conserved LSU130F and LSU559R sequences ([9]; numbering relative to *Escherichia coli *23S rRNA gene). The design goal of this exercise was to construct a bank of 192 barcoded LSU559R primers, while minimizing the number of pyrosequencing flows required to sequence each barcode.

To explore the sequence space of possible barcodes in the context of the required GS-FLX and LSU sequences, multiple *barcrawl *analyses were performed under systematic permutation of the program's parameters. Results and execution times for a subset of the runs are summarized in Table 3. As expected, barcode length was the primary determinant of the size of final barcode pools. Regardless of the parameters, the number of barcodes in an output set typically was ~1% of the size of the starting pool, indicating that most potential barcodes were rejected. For example, the default values produced a pool of 2774 barcodes out of a starting pool of 262,144 sequences (Table 3, run 6). Relative to the default settings, enhancing the stringency of the analysis either by increasing the minimum distance between barcodes (Table 3, run 8) or decreasing the permissible homopolymer length (Table 3, run 13) reduced the final pool sizes by 4-fold or 3-fold, respectively. In comparison, modifying the permissible GC-contents, hairpin lengths, or heteroduplex lengths produced only marginal changes in the extents of final set sizes.

**Table 3:** Barcode sequence space(s) defined by program parameters.

	**Parameters**								**Results**			
		
**Run**	**Len** **-1**	**Min. Dist.** **-m**	**GC Low** **-c**	**GC High** **-g**	**Homo-polymer** **-p**	**Hair-pin** **-a**	**Hetero-duplex** **-b**	**Dels.** **-d**	**Initial Barcode Pool**	**Final Barcode Pool**	**Exec. Time (sec.)^1^**	
											
											**System A^2^**	**System B^3^**
1	4	3	0.3	0.7	3	5	5	1	256	7	0.0 (0.0)	0.0 (0.0)
2	5	3	0.3	0.7	3	5	5	1	1024	26	0.0 (0.0)	0.0 (0.0)
3	6	3	0.3	0.7	3	5	5	1	4096	82	0.4 (0.5)	0.2 (0.4)
4	7	3	0.3	0.7	3	5	5	1	16384	218	3.0 (0.0)	1.2 (0.4)
5	8	3	0.3	0.7	3	5	5	1	65536	760	54 (0.0)	24 (0.4)
**6^4^**	**9**	**3**	**0.3**	**0.7**	**3**	**5**	**5**	**1**	**262144**	**2774**	**978 (0.4)**	**419 (5.4)**
7	9	3	0.3	0.7	3	5	5	0	262144	3113	12 (0.0)	6.0 (0.0)
8	9	4	0.3	0.7	3	5	5	1	262144	593	159 (0.4)	68 (0.8)
9	9	3	0.3	0.7	4	5	5	1	262144	3294	1536 (0.8)	655 (5.8)
10	9	3	0.3	0.7	3	4	4	1	262144	2774	979 (0.7)	416 (1.2)
11	9	3	0.3	0.7	3	6	6	1	262144	2774	977 (1.1)	419 (9.3)
12	9	3	0.4	0.6	3	5	5	1	262144	2155	493 (0.5)	211 (0.8)
13	9	3	0.3	0.7	2	5	5	1	262144	779	42 (0.4)	18 (0.4)
14	9	3	0.2	0.8	3	5	5	1	262144	2914	1153 (0.8)	491 (4.9)
15	9	3	0.1	0.9	3	5	5	1	262144	2910	1177 (0.0)	505 (5.5)
16	10	3	0.3	0.7	3	5	5	1	1.10E+06	9375	16507 (1857)	6650 (23)

^1 ^Elapsed time of execution: Mean (St. Dev) seconds.

^2 ^Executed on a 2 GHz Intel Core Duo MacBook Pro. 1 GB 667 MHz DDR2 SDRAM. Mac OSX version 10.5.4.

^3 ^Executed on a workstation with 2 × 3 GHz Dual-Core Intel Xeon 5160 processors. 12 GB 800 MHz DDR2 FB-DIMM. Fedora Core 5 operating system.

^4 ^Default parameters

**Figure 4:**
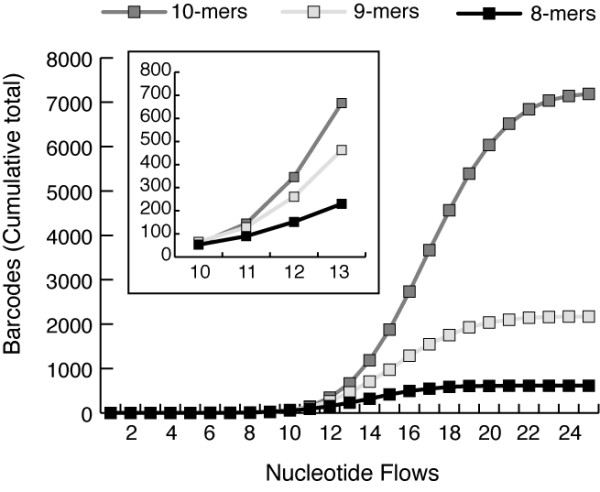
**Cumulative distribution of barcodes as function of pyrosequencing nucleotide flows**. *Barcrawl *analyses were performed for range of barcode lengths. The cumulative sums of selected barcodes are plotted vs. pyrosequencing nucleotide flows. The inset box shows the region of this plot bounded by flows 10-13 and illustrates that about flow #11, the sequence space of 10-mers > 9-mers > 8-mers.

Fig. 4 presents cumulative distribution plots of barcodes as a function of pyrosequencing nucleotide flow, for 8-mers (Table 3, run 5), 9-mers (Table 3, run 6), and 10-mers (Table 3, run 16). As demonstrated in this plot, for pyrosequencing flows greater than 10, the cumulative number of barcodes available at a given nucleotide flow increased with barcode length. For example, in the case of either 9-mer or 10-mer barcodes, the desired sets of 192 barcodes could be selected from barcodes that required <= 12 nucleotide flows. In contrast, a comparable set of 8-mer barcodes would have required selection of barcodes requiring 13 nucleotide flows. Although somewhat counterintuitive, this result indicates that the choice of longer barcode lengths (e.g., 9-mers, 10-mers) can be used to minimize overall sequencing effort (i.e., average number of nucleotide flows) relative to shorter barcodes. In other words, longer barcode lengths can in some circumstances decrease the average number of sequencing flows required to sequence a set of barcoded oligonucleotides.

The default parameters produced a set of 2774 potential barcodes of length 9 nt. Inspection of these results indicated that cutoff values for maximum hairpin and heteroduplex lengths could be made more stringent (i.e. <= 4 basepairs) without compromising the design goal of 192 barcodes. Therefore the parameters specified in Table 3, Run 10 were used to select a set of LSU559R barcoded primers. In this example, *barcrawl *was invoked with the following command:

barcrawl -l 9 -m 3 -p 3 -a 4 -b 4 -c 30 -g 70 -f5 GCCTCCCTCGCGCCATCAG -f3 NNCATTMTACAAAAGGYACGC -r GCCTTGCCAGCCCGCTCAGNNCCGAATGGGGVAACCC -j5 -j3 -d

The starting pool consisted of 262,144 oligonucleotides. Of these, 260,736 oligonucleotides were discarded by application of the GC-content (47,104 oligos culled), homopolymer (58,816), hairpin (65,624), join (14,115), deletion (15,007), and minimum base difference (59,710) filters. The final pool of 1768 acceptable barcodes was sorted in ascending order of 454 GS-FLX flows and oligonucleotides were synthesized for the top 96 barcodes.

**Figure 5:**
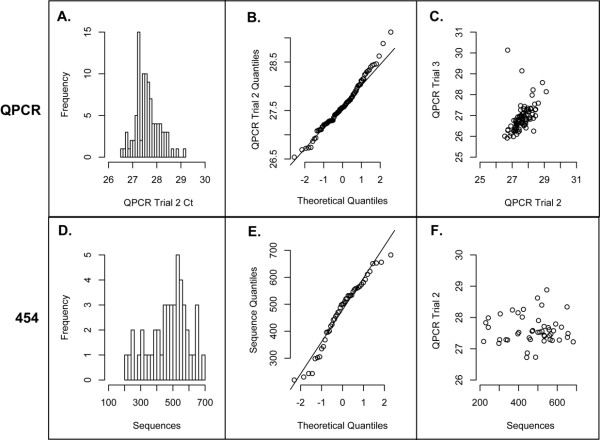
**Functional assessment of barcoded primers by quantitative PCR and pyrosequencing analysis**. A. Distribution of Ct scores for 96 barcoded LSU559 primers tested under identical reaction conditions. Bars represent relative frequency of occurrence of Ct scores. B. Q-Q plot of sample Ct scores versus those drawn from a theoretical normal distribution. Line denotes expected values for data obtained from a normal distribution. C. Scatterplot of Ct scores for each barcoded primer assayed in two separate PCR experiments and using different primer aliquots. The Pearson correlation coefficient of the two data sets is 0.48. B. Distribution of trimmed pyrosequencing reads for each of 47 barcoded LSU559 primers tested under indentical reaction conditions. Bars represent relative frequency of occurrence of sequence lengths (nts). E. Q-Q plot of pyrosequencing read lengths versus those drawn from a theoretical normal distribution. Line denotes expected values for data obtained from a normal distribution. F. Scatterplot of pyrosequencing read lengths vs. Ct scores for each barcoded primer assayed by pyrosequencing. The Pearson correlation coefficient of the two data sets is -0.01.

The relative abilities of the barcoded LSU559R oligonucleotides to prime broad-range PCR were assessed by setting up parallel PCR reactions that used the same cocktail of PCR reagents, metagenomic DNA, and reverse primer. Real-time PCR reactions were conducted and Ct scores measured for each oligonucleotide (Fig. 5). As shown in Figs. 5a and 5b, the distribution of Ct scores was approximately normal. In this experiment, Ct values ranged from 26.5 to 29.1 cycles (mean = 27.6 ± 0.48 cycles). Under perfectly efficient exponential PCR amplification, this would translate to a 6-fold difference (2^2.6^) in yield across the range of Ct scores.

To assess whether the observed differences in amplification within the set of primers was due to factors intrinsic to the barcode sequences or to normal experimental variability, the experiment was duplicated using different metagenomic DNA and newly diluted sets of LSU559R oligos. Fig. 5c presents a comparison of the Ct values obtained for each primer in duplicate experiments. We interpret the Pearson correlation coefficient for these data (r^2 ^= 0.48), as indicating that only 48% of the variability could be attributed to intrinsic differences in the set of barcoded oligonucleotides. This could represent sequence-specific factors or variability in the concentrations of oligonucleotide stocks provided by the manufacturer.

Finally, barcoded LSU559R oligos were tested for their ability to support multiplexed pyrosequencing on the Roche GS-FLX platform. Pre-sequencing PCR amplification, clean-up, and pyrosequencing steps were performed as described in Materials and Methods. A selection of 48 barcoded primers was assayed in order to balance sequencing costs against the need to ensure an adequate distribution of sequences across oligonucleotides; for the purposes of this experiment, we predicted that a 1/8 platform run would provide sufficient sequencing coverage of all 48 oligos (>400 sequences on average).

To mitigate the effects of experimental variability, each barcoded LSU559R primer was tested in triplicate sequencing reactions. Despite this redundancy, one well of the PCR plates (H01) was systematically prone to evaporation during the triplicate experiments. Although earlier experiments demonstrated that the barcoded primer in this well of the microtitre plate was functional, only minimal PCR product was obtained prior to sequencing in this set of experiments. Consequently, the results for this oligo were censored.

Pyrosequencing data were provided in two files containing either FASTA formatted sequences (lsu559R.fa) or their corresponding quality scores (lsu559R.qual). These, along with a file specifying sequences of the 47 barcodes under investigation (lsu559R.bar), were provided as input to *bartab*. Sequence reads were trimmed and barcodes identified using the *bartab *system call: bartab -in lsu599R.fa -qin lsu599R.qual -bar lsu559R.bar -min 175 -out lsu559Rout"). A total of 29,173 raw reads were generated in the 1/8 plate GS-FLX run. From this starting pool, *bartab *output 22,332 polished sequences and rejected 5557 sequences (19%) with length < 175 nt., 814 sequences (2.7%) with ambiguous base calls, and 470 sequences (1.6%) with no valid barcode.

Each of the 47 barcoded LSU559R primers was well-represented in the sequence data set, with a range of 219-693 sequences recovered per barcode. As shown in Figs. 5d and 5e the distribution of sequence counts obtained for each barcode sequence was approximately normal (median = 500.0, mean = 474.7). A scatterplot of the number of sequences generated by barcoded primers vs previously measured QPCR Ct values for the primers showed no correlation between PCR and sequencing efficiencies (Pearson correlation coefficient = -0.01). Thus, no bias was observed in the ability of a primer to support high-throughput sequencing under the protocols employed. We conclude that this set of LSU specific barcoded primers is validated for multiplexed sequencing projects.

### Software Performance Issues

As a rough guide to system requirements, benchmark comparisons for common *barcrawl *and *bartab *tasks are summarized in Tables 3 and 4, respectively. *Barcrawl *stores all processed data in RAM, so performance likely will be limited as much by memory as by raw processor speeds. *Barcrawl *operates in approximately exponential time, *O*(*c*^*n*^), proportional to the length of barcodes. Selection of the 2774 LSU559R barcodes by *barcrawl *required 416 seconds on a Linux workstation (Table 3). Performance was most critically affected by the filter that screened for robustness of barcodes to deletion ("-del" option).

**Table 4:** Performance measures for *bartab*:

			**Execution Time (sec.)^1^**			
**Sequences Processed**	**Split Sequences (-spl option)**	**De-replicate Sequences (-rep option)**	**System A^2^**	**System B^3^**	**System C^4^**	**System D^5^**

400,000	-	-	411 (13)	65 (0.82)	145 (8.6)	40 (0.0)
400,000	+	-	993 (193)	71 (2.07)	178 (3.1)	41 (0.0)
400,000	-	+	434 (5.5)	76 (0.75)	151 (0.8)	47 (1.2)
400,000	+	+	927 (65)	78 (0.75)	197 (9.1)	48 (0.6)
800,000	-	-	900 (4.8)	133 (0.98)	289 (14)	81 (0.0)
800,000	+	-	1909 (225)	142 (3.9)	462 (9.0)	89 (6.8)
800,000	-	+	917 (29)	148 (2.3)	299 (1.4)	97 (2.6)
800,000	+	+	2023 (109)	154 (1.6)	465 (0.5)	106 (10)
1,600,000	-	-	1923 (69)	265 (2.2)	573 (11)	164 (2.0)
1,600,000	+	-	3764 (74)	285 (4.2)	1475 (278)	255 (64)
1,600,000	-	+	1909 (88)	304 (1.7)	623 (15)	197 (7.6)
1,600,000	+	+	4938 (968)	313 (4.3)	1482 (129)	281 (62)
3,200,000	-	-	n.d.	549 (8.4)	1161 (35)	415 (34)
3,200,000	+	-	n.d.	587 (13)	3729 (469)	566 (156)
3,200,000	-	+	n.d.	622 (3.6)	1266 (21)	457 (29)
3,200,000	+	+	n.d.	813 (5.8)	3494 (231)	691 (232)

^1 ^Elapsed time of execution: Mean (St. Dev) seconds.

^2 ^Executed on a 2 GHz Intel Core Duo MacBook Pro. 1 GB 667 MHz DDR2 SDRAM. Mac OSX version 10.5.6.

^3 ^Executed on a 2.6 GHz AMD Phenom 9950 Agena Quad-Core Processor. 4 GB 800 MHz DDR2 FB-DIMM Ubuntu 8.1 operating system.

^4 ^Executed on a Macintosh workstation with 2 × 3 GHz Quad-Core Intel Xeon processors. 8 GB 800 MHz DDR2 FB-DIMM. Mac OS X Server version 10.5.4

^5 ^Executed on a workstation with 2 × 3 GHz Dual-Core Intel Xeon 5160 processors. 12 GB 800 MHz DDR2 FB-DIMM. Fedora Core 5 operating system.

*Bartab *operates in approximately linear time, *O(n)*, proportional to the number of sequences to be processed. In contrast to *barcrawl, bartab *does not store extensive data in RAM; only the file dereplication step stores multiple (unique) sequences in RAM at any given time. A Linux workstation required 40 seconds to trim, annotate, and export 400,000 sequences (Table 4), a comparable number of sequences to that generated in a full run on a GS-FLX pyrosequencer. Dereplication of output files required little additional computational time. In contrast, the overhead required to sort sequences into barcode-specific files was considerable (2× elapsed time) for the Mac OSX operating system. To enhance performance, sequence data can be spread across multiple files. Alternatively, more complex data storage strategies, such as the use of application-specific databases may be implemented in the future.

## Conclusion

The software programs *barcrawl *and *bartab *were developed to expedite the design, optimization, and tracking of barcoded oligonucleotides in projects that require highly multiplexed PCR and DNA sequencing. The software has been used to construct and experimentally validate sets of 192 16S rDNA and 96 23S rDNA primers. Any suggestions for improving the capabilities of this software are welcomed.

## Methods

### Quantitative PCR

The set of 96 barcoded LSU559R oligonucleotides designed in this study were assayed in parallel by quantitative PCR using a DNA Engine Opticon System (Bio-Rad Laboratories, Inc., USA). The forward primer was LSU130F, modified to incorporate the 454 Life Sciences "B" primer (5' GCC TTG CCA GCC CGC TCA GNN CCG AAT GGG GVA ACC C). 25 μl Q-PCR reactions contained 200 nM of each primer, 10 μl PowerSYBR Green PCR Master Mix (Applied Biosystems, USA), 10.5 μl H_2_O and 1 μl of template DNA (mixed community fecal genomic DNA estimated to contain ~10,000 rRNA gene copies per microliter). 2.5 μl of each barcoded oligonuclotides (2 μM) were aliquoted into a 96-well PCR plate. A cocktail of all other reagents was prepared and then dispensed into the PCR plate. The cycling protocol was as follows: 1) initial denaturation at 95°C (6 min); 2) 35 cycles of 92°C (15 sec.), 54°C (15 sec.), 60°C (30 sec), 70°C (1 sec for measurement of fluorescence); and 3) Denaturation curves from 60°C to 95°C.

Following amplification, data were normalized by baseline-subtraction (average over cycles 3-7) and Ct scores exported to Microsoft Excel and R [10] for further analysis. Q-PCR products were analyzed by temperature gradient denaturation profile in order to qualitatively assess reaction product specificity. PCR products generated in the first experiment also were inspected by agarose gel electrophoresis to confirm that amplicons were of the predicted length.

### Pyrosequencing Analysis

Forty-eight barcoded primers (columns 1-6 of a 96-well microtitre plate of diluted primers) were selected for validation by sequencing on a 1/8 GS-FLX platform run. Each 30 μl PCR reaction contained 12 μl 2.5× HotMasterMix (5 PRIME Inc., MD, USA), 0.2 μM LSU130F, 0.2 μl barcoded LSU559R and mixed-community genomic DNA. To standardize reaction components, a single master PCR cocktail containing all reagents other than the barcoded LSU559R primers was set up then aliquoted into a 96-well PCR microtitre plate. Reactions also were performed in triplicate. PCR products were amplified through 35 cycles of 92°C 15 secs, 50°C 15 secs, and 65°C 45 secs. To assess the results of PCR 5 μl aliquot of each reaction was separated by 1.5% agarose gel electrophoresis in Tris/Borate/EDTA buffer. A systematic error caused by evaporation was observed in 2 of 3 replicates for well H01; consequently, the results for this primer were censured.

The goal of the sequencing experiment was to determine whether the selected barcode sequences influence the efficiency of pyrosequencing. To lessen the impact of bias due to differences in template yields submitted to sequencing, quantities of PCR products were normalized by use of the SequalPrep™ Normalization Plate Kit (Invitrogen Inc., CA, USA), following the manufacturer's protocol. Triplicate PCR reactions were pooled prior to normalization. Amplicons were eluted from normalization plates in 30 μl of 10 mM Tris-Cl (pH 8.0). Products were then pooled by combining 25 μl aliquots of each amplicon. This pool was lyophilized to reduce its volume to ~30 μl and then one-half was electrophoresed through a 1.5% agarose gel in Tris/Acetate/EDTA containing ethidium bromide. DNA was visualized by long-wavelength ultraviolet irradiation and a product of the appropriate size excised with a sterile razor blade. DNA was eluted from the gel slice by use of the Montage™ DNA Gel Extraction Kit (Millipore Corp., MA, USA). This pooled DNA was provided to the Colorado Consortium for Comparative Genomics for pyrosequencing on a 454 Life Sciences GS-FLX instrument.

### Statistical Analysis

Results of QPCR and pyrosequencing experiments were analyzed by use of the R statistical package [10].

## Availability and requirements

Project name: *barcrawl/bartab*

Project home page: 

**Operating system**: Platform indendepent

**Programming language**: C++

**Other requirements**: gcc compiler package

**License**: GNU general public license

**Any restrictions to use by non-academics**: None

## Authors' contributions

DNF conceived the study, created the software, performed the experiments, and wrote the manuscript.

## References

[B1] Frank DN, Pace NR (2008). Gastrointestinal microbiology enters the metagenomics era. Curr Opin Gastroenterol.

[B2] Peterson DA, Frank DN, Pace NR, Gordon JI (2008). Metagenomic approaches for defining the pathogenesis of inflammatory bowel diseases. Cell Host Microbe.

[B3] Mardis ER (2008). Next-generation DNA sequencing methods. Annu Rev Genomics Hum Genet.

[B4] Margulies M, Egholm M, Altman WE, Attiya S, Bader JS, Bemben LA, Berka J, Braverman MS, Chen YJ, Chen Z (2005). Genome sequencing in microfabricated high-density picolitre reactors. Nature.

[B5] Parameswaran P, Jalili R, Tao L, Shokralla S, Gharizadeh B, Ronaghi M, Fire AZ (2007). A pyrosequencing-tailored nucleotide barcode design unveils opportunities for large-scale sample multiplexing. Nucleic Acids Res.

[B6] Hamady M, Walker JJ, Harris JK, Gold NJ, Knight R (2008). Error-correcting barcoded primers for pyrosequencing hundreds of samples in multiplex. Nat Methods.

[B7] Meyer M, Stenzel U, Hofreiter M (2008). Parallel tagged sequencing on the 454 platform. Nat Protoc.

[B8] Frank DN (2008). XplorSeq: a software environment for integrated management and phylogenetic analysis of metagenomic sequence data. BMC Bioinformatics.

[B9] Lane DJ, Goodfellow SaM (1991). 16S/23S rRNA sequencing. Nucleic acid techniques in bacterial systematics.

[B10] R Team DC (2005). R: A Language and Environment for Statistical Computing.

